# The Impact of Color Cues on the Learning Performance in Video Lectures

**DOI:** 10.3390/bs14070560

**Published:** 2024-07-02

**Authors:** Linwei She, Zhiguo Wang, Xiaohui Tao, Liqi Lai

**Affiliations:** 1 International Business School, Jinan University, Zhuhai 519070, China; 2 Network and Educational Technology Center, Jinan University, Guangzhou 510632, China; 3 Modern Education Technology Centre, Jinan University, Zhuhai 519070, China; 4 GBA and B&R International Joint Research Center for Smart Logistics, Jinan University, Zhuhai 519070, China

**Keywords:** color cues, video lectures, eye tracking

## Abstract

This study explores the learning effects of color cues in video lectures and their underlying mechanisms. With the rapid growth of online education, lifelong learning, and blended learning, video lectures have become integral to teaching and learning. Color, a crucial element in visual design, directs attention, organizes content, and integrates information. Evaluating 78 college students, we assessed learning performance by comparing video content with no-color, single-color, and multi-color cues using eye-tracking technology and cognitive load scales. Results indicate that students viewing videos with color cues demonstrated better retention and transfer test performance, while absence or excess of color cues increased cognitive load. These findings have practical implications for video producers and provide a theoretical foundation for enhancing learners’ viewing experience and overall effectiveness. This study not only offers an in-depth analysis of color cue utilization in video lectures, highlighting their positive impact on learning outcomes but also introduces fresh perspectives for educational technology and cognitive psychology research. Future investigations should consider color cue effects in diverse cultural contexts and subject areas, exploring varied strategies to optimize the learning experience.

## 1. Introduction

With the high growth of online education, lifelong education, and blended learning, the design and production of video lectures has become a topic of interest for researchers [1]. They are used to enhance traditional classroom courses, blended learning (i.e., a combination of online and traditional classroom learning), and fully online courses [2]. They are powerful learning tools because they can simultaneously present knowledge through vivid images and audio formats. Previous studies have explored the effectiveness of instructional videos in different disciplines, such as mathematics, medicine, and computing [3,4,5].

The attentional guidance (or selection function) of clues refers to directing learners’ attention to specific locations and information (important information useful for understanding principles and constructing mental representations) in multimedia [6,7]. This idea appeared earlier in the study by Mautone and Mayer [8] (Experiment 1). In their experiment, they tested various cue formats (introductory, conjunctive, bolded, italicized) and found that cues had a directing effect on attention and that the cued group had higher learning performance. Later, Bétrancourt [9] proposed the concept of the attention-guiding principle to highlight the importance of cues in multimedia learning and suggested the use of visual cues to guide learners’ attention to important content in the learning material.

Color is an important element that influences learning and experience in video lectures [10]. However, we found that previous studies have focused more on the color coding of picture text and also on individual colors compared to gray or no color, and few have compared the effects between monochrome, multicolor, and no color, or have not studied learning performance, gaze duration, and cognitive load simultaneously. The use of color in the design of instructional materials not only attracts and maintains learners’ attention, but also helps them better organize and remember information.

However, Although the role of color has been extensively studied in printed materials and static slides, how color cues affect learners’ cognitive load and learning performance in dynamic video lecture environments has not been fully explored. This study aims to fill this research gap by investigating the effects of color cues on learner efficiency using eye-tracking techniques and cognitive load scales. Using a single-factor between-subjects design, we compared colorless, monochromatic, and multi-colored cues in video lectures, aiming to provide practical insights into the experiences and outcomes of video designers, video producers, and learners.

## 2. Theoretical Background and Literature Review

### 2.1. Cognitive Load Theory and Multimedia Learning Theory

Cognitive Load Theory and Multimedia Learning Theory are two important theoretical frameworks that guide instructional design and the development of learning materials [11,12]. Cognitive Load Theory focuses on reducing learners’ cognitive load through instructional design to promote effective learning. Multimedia Learning Theory, on the other hand, emphasizes the use of multimedia resources and technological tools to optimize the learning process and enhance learning efficiency.

The core idea of Cognitive Load Theory is the limited capacity of working memory. Working memory consists of visual and auditory processing units, which, together with the larger capacity of long-term memory, are responsible for storing and understanding information [13]. When the design of learning materials exceeds an individual’s working memory capacity, it can negatively impact learning outcomes. Therefore, Cognitive Load Theory emphasizes the importance of designing instructional strategies to control cognitive load, such as using advance organizers and presenting materials in segments, to reduce intrinsic cognitive load [14]. Additionally, simplifying material content can reduce extraneous cognitive load.

Multimedia Learning Theory is based on the dual-channel assumption, which suggests that humans have two independent systems for processing visual and verbal mate-rials [15]. This theory argues that effectively utilizing these two channels can enhance learners’ information processing and knowledge construction. For example, presenting images and textual descriptions simultaneously can help learners establish better connections between concepts, thereby improving learning outcomes. However, multimedia learning also carries the risk of cognitive overload, where learners’ cognitive load exceeds their processing capacity, hindering effective learning.

In recent years, researchers have attempted to integrate Cognitive Load Theory and Multimedia Learning Theory to further optimize instructional design in multimedia learning environments [16,17]. For instance, studies have shown that appropriately increasing metacognitive monitoring activities in multimedia learning can enhance transfer effects, despite increasing overall cognitive load. Furthermore, research has also found that instructional explanations and self-explanation prompts can modulate related cognitive load during learning with materials of different difficulty levels, thereby influencing learning outcomes.

### 2.2. Related Research on Color in Multimedia Learning

With the development of information technology, video lectures have become an integral part of the educational field. By combining visual and auditory elements, video lectures provide learners with a rich and dynamic learning experience. However, how to design effective video lectures to facilitate learners’ cognitive processes and enhance learning effectiveness remains an important topic in educational research.

In the context of multimedia learning theory, color, as a powerful visual tool, has been shown to have a significant impact on learners’ attention guidance and information organization. Research in multimedia learning has emphasized the importance of design elements such as color, animation, and highlighting that can help learners better understand and remember instructional content [18]. Color not only enhances visual salience, but also serves as a framework for organizing information and helps learners structure their knowledge [19].

Despite the extensive research on the use of color in multimedia learning, there is still relatively limited research on the specific role of color in video lectures, especially in online and blended learning environments. A study by An and Li [20] explored the effect of PPT background color on learning outcomes and found that PPTs with white backgrounds were the most suitable for learning, while those with blue backgrounds were the least effective.

Furthermore, with the increasing use of video conferencing technology in education, especially during the COVID-19 epidemic, learner acceptance and experience of using videoconferencing tools has become a hot topic of research [21]. This requires us to revisit the design of video lectures with various visual elements including the use of colors to adapt to the new learning paradigm and learners’ needs.

Color, in particular, has been extensively studied as a cueing mechanism. Ozcelik et al. [22] used color as a cue and recorded eye movements during learning and found that the subjects looked at the relevant information more often and for a longer total time. Boucheix and Lowe [23] found that when dynamic color was used as a cue, subjects focused more and longer on areas of interest that were low in visual salience but high in relevance to the learning topic, suggesting that learners may have ignored the high visual salience and focused more on areas of interest related to the learning topic. Liu et al. [24] showed that color coding designs were more beneficial than grayscale designs, as evidenced by smaller pupil diameter, shorter gaze duration, higher EEG theta, and alpha band power, lower EEG cognitive load, and better learning performance. Erol Ozcelik et al. [25] showed that color coding improved memory retention and transfer performance. The facilitation of learning by color coding is due to the efficiency of finding the corresponding information between illustrations and text. Color coding also draws learners’ attention to perceptually salient information. Kalyuga et al. [26] demonstrated that learners who learned color-coded formats scored higher on multiple-choice tests than learners who learned traditional formats. Similarly, Keller et al. [27] found that students scored higher on knowledge tests when the information visualization was color coded than when it was monochrome coded. These results suggest that color coding enhances learning. An L and Li Z [20] evaluated color psychology, combined with questionnaires on a five-point scale, and used 15 college students as subjects to record their reading of PPTs with different color backgrounds and analyze the influence of background color on learners. It was found that white was most suitable as the background color of PPT, followed by yellow, while blue was least suitable as the background color.

Color, as a prominent design element in multimedia learning environments, influences cognitive and affective processes. It is believed that color can increase learners’ motivation by enhancing the visual appeal of the learning environment and, to some extent, guide learners’ attention during the learning process [28]. Color can also influence the level of cognitive processing in learning. Research on the cognitive effects of color in multimedia learning focuses on using color as a signaling cue to highlight essential elements of the learning material, thereby directing learners’ attention to them [18,29].

Color is an important element that influences learning and experience in video lectures [30]. However, through the above literature review, we found that previous studies have focused more on the color coding of picture text and also on individual colors compared to gray or no color, and few have compared the effects between monochrome, multicolor, and no color, or have not studied learning performance, gaze duration, and cognitive load simultaneously. Therefore, the present study compared the effects of no-color cues, single-color cues, and multi-color cues in video lectures through three experimental conditions. The single-color color cue consisted of blue as the color block highlighting the focal content, whereas the multi-color cue had red and blue as the color block highlighting the focal content. The no-color cue did not show any cue as a control.

## 3. Research Question and Hypotheses

Against this background, this study poses the following research question: how do color cues affect learners’ cognitive load and learning performance in video lectures? We hypothesize that color cues can enhance learning by directing attention and organizing information, but the use of colors needs to be moderate, and excessive or inappropriate use of colors may increase cognitive load.

To explore this hypothesis, this study will assess learner performance under different color cueing conditions using eye-tracking technology and cognitive load scales. We anticipate that appropriate color use will help improve learner retention and transfer, while inappropriate color use may negatively affect learning effectiveness. Based on the aforementioned literature review, the following hypotheses are formulated for the present study:

(1) Learning performance test scores will be highest in the multi-colored cue condition, followed by the single-colored cue condition, and lowest in the no-colored cue condition.
(2) The length of gaze on Areas of Interest (AOIs) will be longest in the multi-colored cue condition, followed by the single-colored cue condition, and shortest in the no-colored cue condition.
(3) Cognitive load scale test scores will be lowest in the multi-color cue condition, higher in the single-color cue condition, and highest in the no-color cue condition.

## 4. Methods

### 4.1. Participants and Design

This study employed random sampling methods to ensure the representativeness and reliability of the sample. Participants were 78 undergraduate and graduate students (34 males and 44 females) who were recruited from a university in China primarily through an electronic promotional poster. Their ages ranged from 18 to 26 years old (M = 19.603, SD = 1.523). According to information provided in their informal interviews prior to the experiment, participants had a wide range of majors (e.g., electrical engineering and its automation, financial engineering, artificial intelligence, translation, sociology, industrial engineering, and business administration), but none were familiar with the topics presented in the video handouts. All were native Chinese speakers, and none were colorblind. All participants were informed of the purpose and requirements of the experiment in advance and received a bottle of drink as a reward at the end of the experiment.

This study employed a single-factor between-subjects design. Participants were randomly assigned to one of three groups: no-color cue (*n* = 26), single-color cue (*n* = 26), and multi-color cue (*n* = 26). This structure facilitates the examination of how different color cue conditions affect learning performance and cognitive load.

### 4.2. Apparatus

Eye movements were recorded with a Tobii Pro Spectrum eye-tracking device (Tobii AB Ltd., Danderyd, Sweden). Each participant, before watching the experimental video, followed the on-screen prompts for the eye-tracking device calibration in two steps. The display had a resolution of 1980 × 1080 pixels and a refresh rate of 60 Hz. Participants received audio information through headphones.

### 4.3. Materials

#### 4.3.1. Stimuli

**Figure 1:**
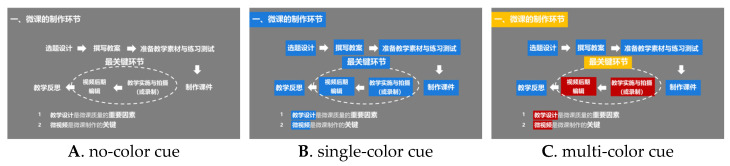
Screenshots of each condition of the three micro-video species.

Three types of micro-videos were used, each lasting about 5 min. The micro-video content was about the development methods and techniques of micro-lessons, all of which have no teacher present and were presented in the form of text, pictures and shapes. The three forms of micro-video are shown in Figure 1: (1) No-color cue condition: the screens were presented in the form of text and pictures, and the key content was not highlighted with color cues. (2) Single-color clue condition: the screen is displayed in the form of text and pictures, and the key content is highlighted with the same color clue. (3) Multi-color clue condition: the picture is displayed in the form of text and pictures, and the key content is highlighted by many different color clues.

#### 4.3.2. Measurement

Demographic questionnaire: each participant was asked to fill in gender, grade, major, and age. Demographic data was collected as a control factor for the study.

##### Priori Knowledge Test

Eight single-choice questions were used to test participants’ a priori knowledge of microlearning. These questions were asked by the teachers in the study. There were no questions on specific learning topics to avoid any influence of expectations on learning performance (e.g., David Penrose of San Juan College in New Mexico, USA, is known as? A. the “godfather” of the upside-down classroom B. the “2-min professor” C. the “1-min professor” D. a pioneer; which of the following is not a classification of the content of knowledge by the way it is taught? A. Experimental B. Problem solving C. Lesson presentation D. Lecture-based). Each question has four choices, and only one option is correct. The test score is calculated by assigning 1 point to each correct answer. The maximum total score was 8 points. ANOVA was performed on the prior knowledge and the results are shown in Table 1. From the table, we can see that there was no significant difference between the no-color cue condition, the single-color cue condition, and the multi-color cue condition (*F* = 0.137, *p* = 0.873 > 0.05).

##### Learning Performance Test

The test was developed by the lecturers in the study. All items were derived from the learning process presented in the video lectures. It contains two parts. (1) Retention test: The first part contains six single-choice questions, each with four choices, only one of which is correct, and each correct answer is given 1 point, up to a maximum of 6 points. The three fill-in-the-blank questions contained seven spaces, and each correct answer was given 2 points, with a maximum score of 14 points. In the retention test evaluation, higher scores indicate better retention of the learning content, while the opposite indicates poor mastery of the learning content. The test questions had a satisfactory internal consistency reliability (Cronbach’s α = 0.66). (2) Transfer test: the second part consisted of two open-ended questions that tested participants’ ability to transfer knowledge to a new context: “If an online course needs to be filmed for advanced mathematics, which development method presented in which video do you think would be better to use? Why?” and “What are the post-processing aspects of the video? Which is the most important one and why?” The scorers then scored the answers to the first question in two dimensions: the best way to develop (5 points) and the reason (5 points), and the scorers scored the second question in three dimensions: the post-production of the video included aspects (4 points), the most important aspects (3 points) and the reason (3 points). Each question scored 10 out of 20. Two quiz questions were scored independently by two raters, and the average of both was taken as the final quiz score. The analysis revealed that the agreement between the two raters was high (Cronbach’s α = 0.97, 0.96) and the test had high internal consistency reliability (Cronbach’s α = 0.75).

##### Cognitive Load Measure Test

Cognitive load was measured by a 9-point scale of mental effort developed by Paas [31], ranging from “very, very low effort” to “very, very high effort”. This scale is widely used in research, and mental effort can be considered to reflect the cognitive load of actuarial work [32].

### 4.4. Analysis of Oculomotor Data

Participants’ visual perception was measured by an eye-tracking technique. This technique is considered to be a promising tool for tracking visual attention because participants’ eye movements (e.g., gaze, sweep, blink) can be recorded in real-time. Eye movements reflect visual attraction allocation. Previous studies have shown a positive correlation between learners’ gaze and visual attention; the longer the gaze is on an area, the more visual attention is given to that area [33]. Therefore, in this study, in order to analyze the effect of color cues on students’ visual search and attention allocation, we calculated the total gaze duration of 10 areas of interest (AOIs) in the video frame of the production session of the micro-lesson in the instructional video, where all the content was focused and color blocks appeared in the color cue condition and not in the no color cue condition.

### 4.5. Procedure

The study was conducted in the laboratory and took approximately 20 min. First, all participants completed an a priori knowledge test and were then escorted to the eye-tracking room. Participants were randomly assigned to one of three conditions: the no-color cue condition, the single-color cue condition, and the multi-color cue condition. Participants watched the video lectures individually, without pauses, and completed retention and conversion tests, as well as a cognitive load test immediately after viewing.

## 5. Results

Analyses of variance (ANOVAs) of the experimental conditions (no-color cue, single-color cue versus multi-color cue) as between-subjects factors were conducted on the scores of the learning performance tests (Retention and Transfer), the total gaze duration, and the scores of the cognitive load scales, as shown in Table 2.

### 5.1. Retention

**Figure 2:**
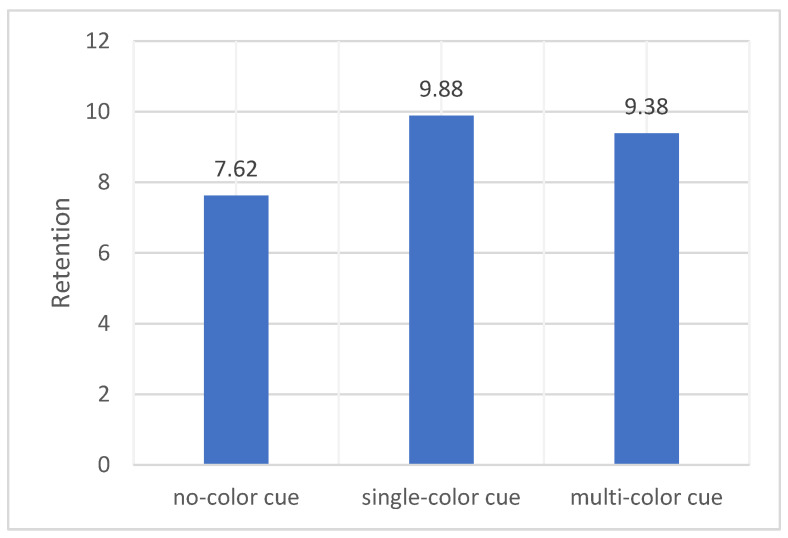
Differences in retention across the three conditions.

The means and standard deviations of the participants’ retention are shown in Table 2. There were no significant differences in student memory test scores between the three conditions (*F* (2, 75) = 2.694, *p* = 0.074). However, participants who watched the instructional video with the color cues condition performed significantly higher than the memory test scores of participants without color cues (see Figure 2).

### 5.2. Transfer

**Figure 3:**
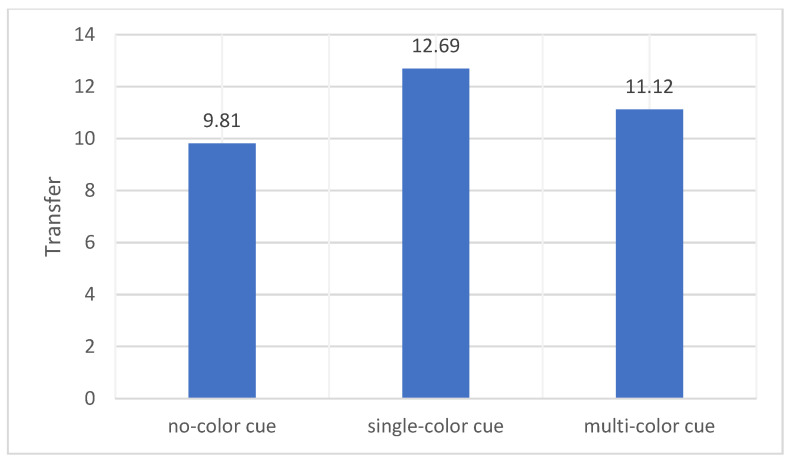
Differences in transfer across the three conditions.

The means and standard deviations of the participants’ transfer are shown in Table 2. There were also no significant differences in student transfer scores across the three conditions (*F* (2, 75) = 2.523, *p* = 0.087). However, participants who watched the instructional video with the color cues condition performed significantly higher than the memory test scores of participants without color cues (see Figure 3).

From the above analysis, we can find that for both retention scores and transfer scores, the results partially supported our first hypothesis. Participants learned better with video lectures that had color, but single color worked better than multicolor.

### 5.3. Visual Attention

**Figure 4:**
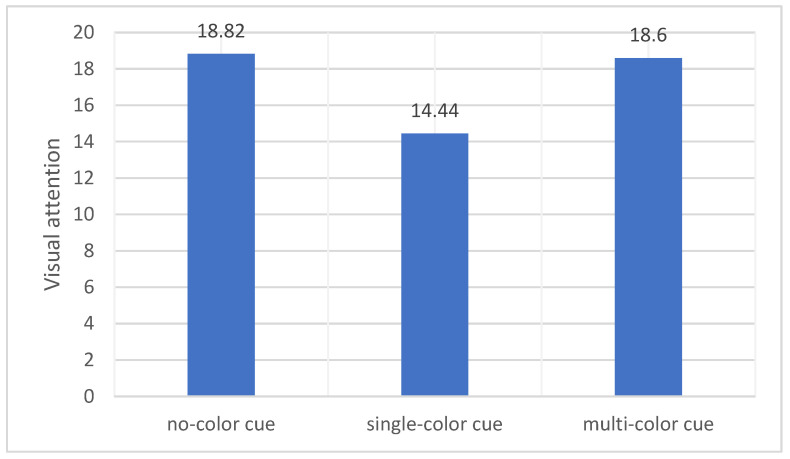
Differences in total fixation duration across the three conditions.

The means and standard deviations of the participants’ total fixation duration are shown in Table 2. No significant effect of attention span was found here (*F* (2, 75) = 2.735, *p* = 0.071). No color is higher than multicolor and single color, and the results partially support our second hypothesis (see Figure 4).

### 5.4. Cognitive Load

**Figure 5:**
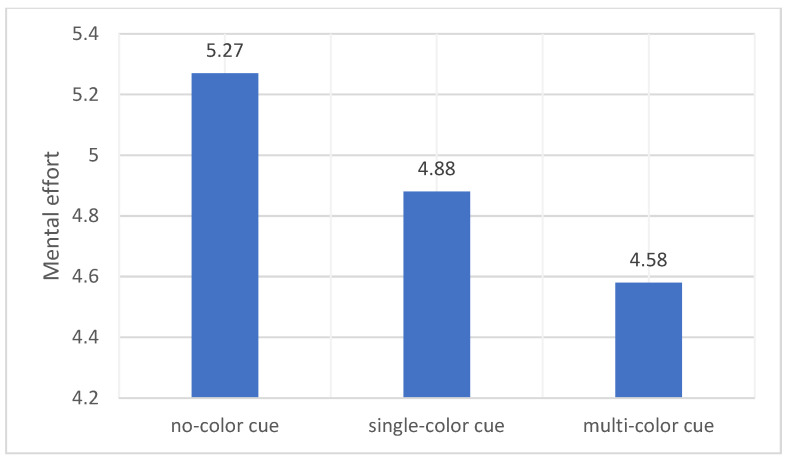
Differences in mental effort across the three conditions.

The means and standard deviations of the participants’ mental effort are shown in Table 2. No significant effect of attention span was found here (*F* (2, 75) = 0.637, *p* = 0.532). The results are consistent with our third hypothesis; the mental effort involved in tasks with no color, single color, and multiple colors gradually decreases (see Figure 5).

## 6. Discussion

The necessity to adopt eye-tracking technology in this study stems from its ability to provide precise measurements of where and how long learners look at different parts of the video. This data is crucial for understanding how color cues influence attention distribution and cognitive load. Eye-tracking allows us to explore the underlying mechanisms of learning processes that are not visible through traditional assessment methods.

This study examined whether color cue pairs were superior to no color cue cues for facilitating learning in video lectures, and if such an effect did exist, what the potential mechanisms for this effect might be. The results showed that participants who viewed color cues showed higher academic performance compared to students who viewed no-color cue cues, while those who viewed single-color cues showed higher performance than those who viewed multiple color cues. In terms of visual attention, multi-color cues were higher than single-color and no-color cues, while no-color was higher than single-color. Moreover, in terms of cognitive load, single color was the lowest, and multicolor and no color were the same. This suggests that color cues play a greater role in facilitating learning than colorless cues and that single-color cues are superior to multi-color cues. From a practical point of view, it is important to study the effect of color cues in video lectures, where often colors are widely used. In the remainder of this discussion, we will discuss the learning context and cultural background of the experimental implementation, as well as the potential mechanisms by which color cues produce such positive effects.

The results of this study are based on video lecture instructional educational technology, and thus the color cueing effect may not be the same across different knowledge types and different disciplines of video lectures. In addition, the participants of the study were Chinese, and cultural studies have shown that people from different cultures have different cognitive processing styles. Chinese are more holistic and focus more on contextual information than Westerners, whereas Americans are more analytical and focus more on focal information [34,35]. In video lectures, there is also focal information and contextual information. The focal information is the content of the corresponding lecture in the PPT slides, and the contextual information is the rest of the content. In this respect, Chinese people may handle learning information in video lectures differently from Westerners. Thus, attentional cues in video lectures may have different effects and draw different conclusions between Westerners and Chinese.

Some researchers have concluded that there is no effect on whether color can improve learning performance [36,37], and others have concluded that there is an effect [38]. With the current study, our research is consistent with the former, and participants who watched with color cues learned better compared to those who watched without color cues. The review clearly shows that color can carry important meaning and can have an important impact on people’s effect, cognition, and behavior [39].

The present study found that having color cues had a more positive effect than not having color cue cues in improving learners’ academic performance and directing visual attention. This suggests that color cues facilitate learning. This study adds direct empirical evidence to the existing research base, suggesting that adding color, can effectively direct learners’ visual attention to the key content in the video to facilitate learning, but not too much color and not too many colors, otherwise it may have the opposite effect and increase learners’ cognitive load.

We have the following suggestions for the production and optimization of video lectures:

(1) Development of content standards for educational videos: It is recommended that the education sector and relevant institutions develop a set of standards for the design of educational video content, including guiding principles for the use of color. These principles should emphasize that color cues should be used to enhance learners’ understanding and retention and that overuse or inappropriate color combinations should be avoided to minimize unnecessary increases in students’ cognitive load.
(2) Video production training and certification: It is recommended that a training program be developed specifically for educational video production, focusing on effective visual design techniques, including the use of color, optimization of layout, and best practices for presenting information. Producers who complete the training can receive appropriate certification to ensure the quality of educational videos.
(3) Personalize the learning experience for learners: Encourage the development and use of educational video platforms that can be personalized to learners’ preferences and needs. For example, allowing learners to choose different color themes based on their color preferences or adjusting the prominence of color cues to improve learning efficiency and experience.
(4) Cross-cultural research and adaptive design: Given that learners from different cultures may perceive and respond to colors differently, more cross-cultural research is recommended to understand the meaning and impact of colors in different cultures. Based on the results of these studies, educational videos should be adaptively designed to meet the needs of learners from different cultures.
(5) Teacher professional development: It is recommended that the use of color in instructional design be incorporated into teacher professional development courses to help teachers better understand and use color cues to enhance classroom instruction and video lectures.
(6) Learner feedback mechanism: It is recommended that educational institutions establish an effective learner feedback mechanism to collect students’ feedback on the design of educational video content, especially on the use of color, to continuously optimize and improve the video content.

Although this study yielded some results, there are some limitations. First, the sample size. The sample size of this study contains only 78 participants, which is relatively small, and there is a possibility of some bias in the results. Second, the type of knowledge. The types of knowledge are divided into declarative and procedural knowledge [40], including this experiment only on declarative knowledge and not on procedural knowledge, and the difference in the types of knowledge may affect the difference in the results. Limitations of the study method: although pre- and post-measures of knowledge content, cognitive load scales, and eye-tracking have been used, tools such as brain waves have not been used for measurement.

## 7. Conclusions

This study compared the effects of no-color, single-color, and multi-color cues in video lectures. The results showed that color cues were better than colorless cues in improving learning performance, but the difference was not significant in monochrome and multicolor. Also, in terms of cognitive load, too many colors should not be present in the video instruction, otherwise, it will affect the cognitive load of students.

We suggest that future studies should pay attention to the following points: First, future studies should examine whether the effects of color are the same across subject types. Second, the volunteers in this study were all Chinese students and future research should examine and confirm whether color cues have the same effect on the school performance of learners from different cultures in video lectures. Finally, future studies should measure cognitive load using instruments that are more sensitive than self-report measures.

## Figures and Tables

**Table 1:** Results of ANOVA for a priori knowledge scores.

	Mean ± Standard Deviation			*F*	*p*
	No-Color Cue (*n* = 26)	Single-Color Cue (*n* = 26)	Multi-Color Cue (*n* = 26)		
prior knowledge	3.85 ± 1.74	3.62 ± 1.50	3.69 ± 1.62	0.137	0.873

**Table 2:** Means and standard deviations of all variables.

Dependent Variable	No-Color Cue (*n* = 26)		Single-Color Cue (*n* = 26)		Multi-Color Cue (*n* = 26)	
	M	SD	M	SD	M	SD
Retention	7.62	3.50	9.88	3.59	9.38	4.00
Transfer	9.81	5.48	12.69	3.59	11.12	4.65
Visual attention (total fixation duration)	18.82	7.62	14.44	7.93	18.60	7.52
Cognitive load (mental effort)	5.27	2.68	4.88	2.01	4.58	1.88

## Data Availability

The data presented in this study are available upon request from the corresponding author.
